# Ethyl (*E*)-3-(anthracen-9-yl)prop-2-enoate

**DOI:** 10.1107/S1600536812051033

**Published:** 2012-12-22

**Authors:** Bernhard Bugenhagen, Yosef Al Jasem, Bassam al Hindawi, Nathir Al Rawashdeh, Thies Thiemann

**Affiliations:** a Institute of Inorganic Chemistry, University of Hamburg, Hamburg, Germany; b Department of Chemical Engineering, United Arab Emirates University, Al Ain, Abu Dhabi, United Arab Emirates; c Department of Chemistry, United Arab Emirates University, AL Ain, Abu Dhabi, United Arab Emirates

## Abstract

In the asymmetric unit of the title compound, C_19_H_16_O_2_, there are two symmetry-independent mol­ecules (*A* and *B*) that differ in the conformation of the ester eth­oxy group. In the crystal, the mol­ecules form inversion dimers *via* pairs of C—H⋯O inter­actions. Within the dimers, the anthracenyl units have inter­planar distances of 0.528 (2) and 0.479 (2) Å for dimers of mol­ecules *A* and *B*, respectively. Another short C—H⋯O contact between symmetry-independent dimers links them into columns parallel to [10-1]. These columns are arranged into (111) layers and there are π–π stacking inter­actions [centroid–centroid distances = 3.6446 (15) and 3.6531 (15) Å] between the anthracenyl units from the neighbouring columns. In addition, there are C—H⋯π inter­actions between the anthracenyl unit of dimers *A* and dimers *B* within the same column.

## Related literature

For an analogous preparation of the title compound, see: Nguyen & Weizman (2007 ▶). For modeling of the title compound at the B3LYP/6–31G* level, see: Coleman (2007 ▶). For crystal structures of photodimerizable aryl­enes, see: Vishnumurthy *et al.* (2002 ▶); Mascitti & Corey (2006 ▶); Sonoda (2011 ▶); Schmidt (1964 ▶). For the photodimerization of anthracenes in the crystal, see: Schmidt (1971 ▶); Ihmels *et al.* (2000 ▶).
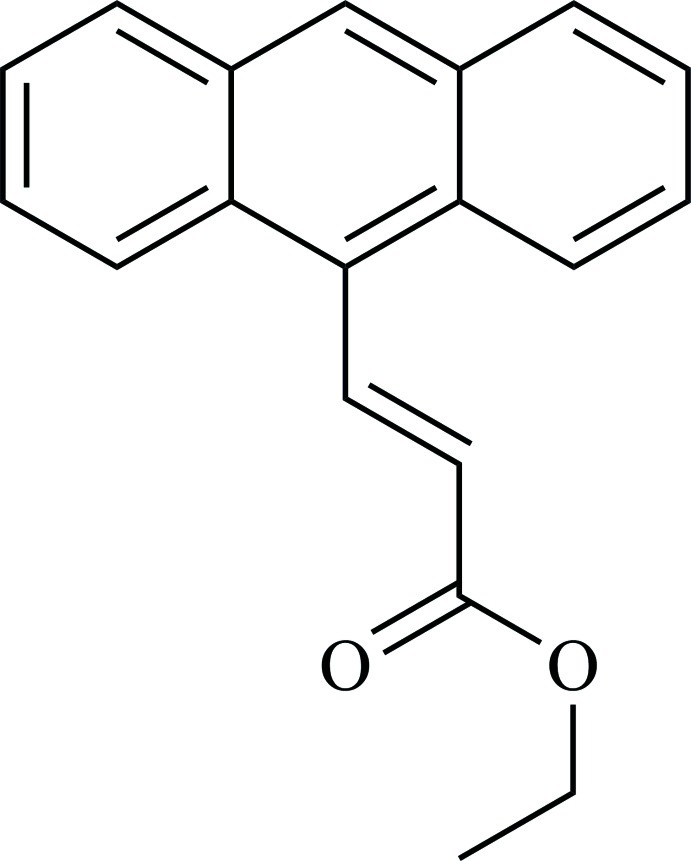


## Experimental

### 

#### Crystal data

C_19_H_16_O_2_
*M* *_r_* = 276.32
Triclinic, 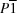

*a* = 8.8700 (5) Å
*b* = 12.8918 (7) Å
*c* = 13.1062 (7) Å
α = 84.389 (4)°
β = 84.620 (4)°
γ = 70.771 (5)°
*V* = 1405.28 (13) Å^3^
*Z* = 4
Cu *K*α radiation
μ = 0.66 mm^−1^
*T* = 291 K
0.22 × 0.11 × 0.09 mm

#### Data collection

Agilent SuperNova Dual Atlas diffractometer
Absorption correction: Gaussian (*CrysAlis PRO*; Agilent, 2012 ▶) *T*_min_ = 0.889, *T*_max_ = 0.942
11350 measured reflections
4901 independent reflections
4250 reflections with *I* > 2σ(*I*)
*R*_int_ = 0.025

#### Refinement

*R*[*F* ^2^ > 2σ(*F* ^2^)] = 0.057
*wR*(*F* ^2^) = 0.171
*S* = 1.10
4901 reflections
381 parameters
H-atom parameters constrained
Δρ_max_ = 0.29 e Å^−3^
Δρ_min_ = −0.26 e Å^−3^

### 

Data collection: *CrysAlis PRO* (Agilent, 2012 ▶); cell refinement: *CrysAlis PRO*; data reduction: *CrysAlis PRO*; program(s) used to solve structure: *SHELXS97* (Sheldrick, 2008 ▶); program(s) used to refine structure: *SHELXL97* (Sheldrick, 2008 ▶) within *OLEX2* (Dolomanov *et al.*, 2009 ▶); molecular graphics: *PLATON* (Spek, 2009 ▶) and *Mercury* (Macrae *et al.*, 2008 ▶); software used to prepare material for publication: *SHELXL97* and *PLATON*.

## Supplementary Material

Crystal structure: contains datablock(s) global, I. DOI: 10.1107/S1600536812051033/gk2545sup1.cif

Structure factors: contains datablock(s) I. DOI: 10.1107/S1600536812051033/gk2545Isup2.hkl

Supplementary material file. DOI: 10.1107/S1600536812051033/gk2545Isup3.cml

Additional supplementary materials:  crystallographic information; 3D view; checkCIF report

## Figures and Tables

**Table 1:** Hydrogen-bond geometry (Å, °) *Cg*1 and *Cg*2 are the centroids of the C1*A*/C2*A*/C7*A*–C9*A*/C14*A* and C2*A*—C7*A* rings, respectively.

*D*—H⋯*A*	*D*—H	H⋯*A*	*D*⋯*A*	*D*—H⋯*A*
C13*A*—H13*A*⋯O1*A* ^i^	0.93	2.56	3.455 (3)	163
C18*B*—H18*B*⋯O2*A* ^ii^	0.97	2.56	3.422 (3)	148
C3*B*—H3*B*⋯O1*B* ^iii^	0.93	2.57	3.470 (3)	162
C6*A*—H6*A*⋯O2*B* ^iv^	0.93	2.67	3.438 (3)	140
C19*A*—H19*E*⋯O1*B* ^v^	0.96	2.66	3.409 (3)	135
C6*B*—H6*B*⋯*Cg*1^vi^	0.93	2.81	3.447 (3)	126
C8*B*—H8*B*⋯*Cg*2^vi^	0.93	2.82	3.439 (3)	124

Symmetry codes: (i) 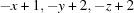; (ii) 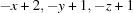; (iii) 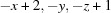; (iv) 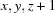; (v) 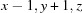; (vi) 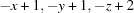.

**Fig. 1.**
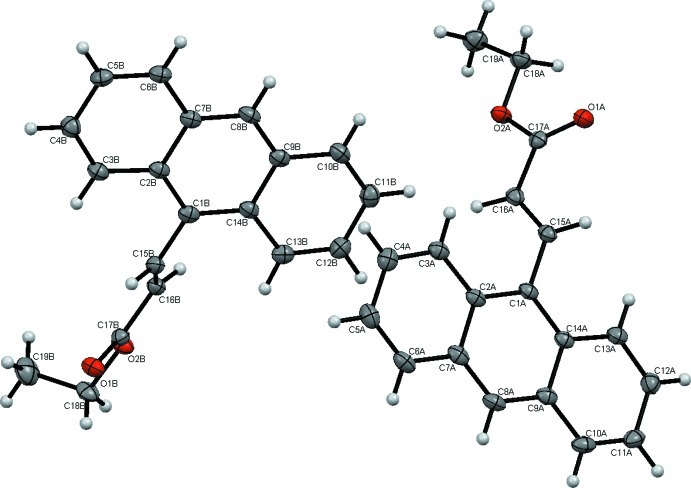
A view of molecules A and B of the title compound with the atom-numbering scheme. Displacement ellipsoids are shown at the 50% probability level.

**Fig. 2.**
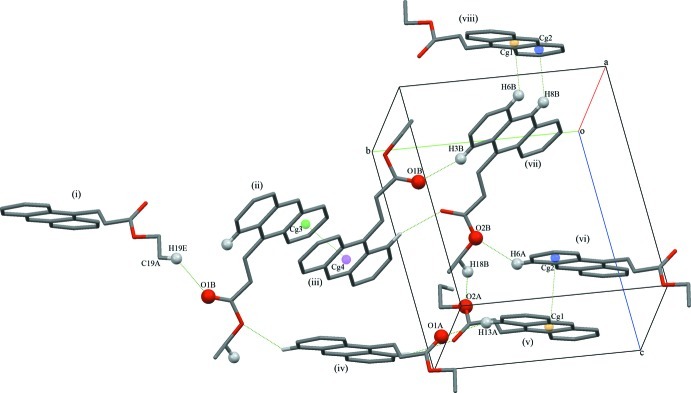
Intermolecular C—H···O, C—H···π, and π–π contacts between molecules of the title compound. [Symmetry codes: (i) -x,3 - y,1 - z; (ii) 1 - x,2 - y,1 - z; (iii) x,1 + y,z; (iv) 1 - x,2 - y,2 - z; (v) x,y,z; (vi) 2 - x,1 - y,2 - z; (vii) 2 - x,1 - y,1 - z; (viii) 1 + x,y,-1 + z]

**Fig. 3.**
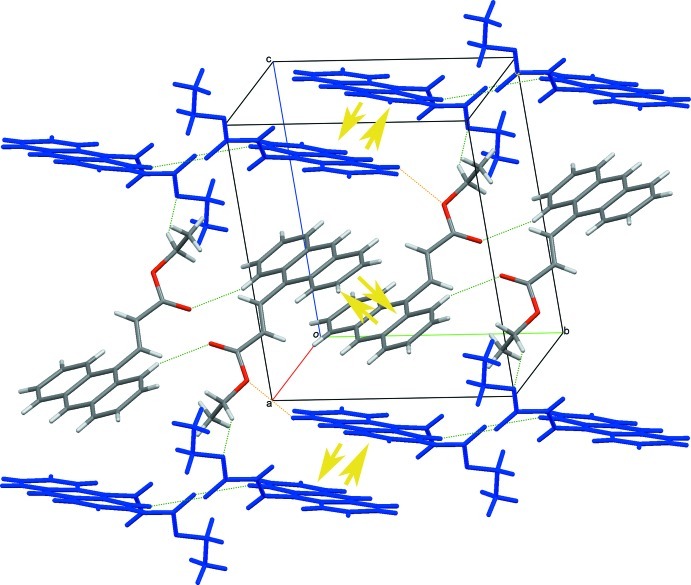
The crystal packing diagram showing the C—H···O intermolecular interactions (orange colored) and π–π stacking interactions between anthracenyl units of neighbouring [1 0 -1] columns indicated by yellow arrows. The A molecules are shown in blue.

## supplementary crystallographic information

### Comment

In our endeavor to carry out [2 + 2]-photocycloaddition of ethyl 3(E)-(9-anthracenyl)propenoate in the solid state, the authors grew single crystals of the title compound to identify intermolecular interactions of the molecule in the crystal, which could control the photocycloaddition (Sonoda, 2011; Schmidt, 1964). In the title compound, the alkyl group forms very different torsion angles with the carboxyl group of the ester function (C17—O2—C18—C19) in molecules A and B, respectively, namely of 178.3 (2) ° in molecule A, and of 87.3 (3) ° in molecule B. Pairs of molecules A and B respectively, are formed by C13A—H13A···O1A (Figure 2) close contact for pairs A, and by C3B—H3B···O1B close contact (Table 1) for pairs B, and with the ring planes of the anthracenyl units of the respective pairs in parallel, but at an offset of 0.528 (2) Å for molecules A and 0.479 (2) Å for molecules B. Pairs A and pairs B interact with each other by C18B—H18B···O2A close contact (Figure 2) to for the [10-1] column. Also, C6B—H6B··· π and C8B—H8B···π interactions (Figure 2) are formed between the pairs B and A in the column. Neighboring columns arranged into [111] layer show partial intercalation to form π–π interaction (Table 1) between the parallel anthracenyl units of the same molecules (A—A and B—B).

The double bonds of two molecules in one pair are aligned parallel to each other at a distance of 5.549 (3) Å for A and 5.627 (3) Å for B. This intermolecular distance between the olefinic moieties is larger than in many of those found for aryl-enes that undergo [2 + 2]-photodimerization readily (Vishnumurthy *et al.* 2002; Mascitti *et al.* 2006). However, the anthracenyl units are aligned parallel to each other with an interplanar distance (C1-C8) of 3.945 (3) Å for A molecules and 4.031 (3) Å for B molecules. This distance lies within the distance of less than 4.2 Å, reported for anthracenes in the crystal that undergo photodimerisation (Schmidt, 1971; Ihmels *et al.*, 2000).

### Experimental

A solventless mixture of 9-anthracenylcarbaldehyde (1.00 g, 4.85 mmol) and ethoxycarbonylmethylidenephosphorane (2.70 g, 7.76 mmol) is heated at 130°C for 3 h. Thereafter, an additional amount of phosphorane (1.00 g, 2.87 mmol) is added and the reaction mixture heated for another hour at 135°C. The cooled solution is subjected directly to column chromatography on silica gel (eluent: *M^t^*BE/CHCl_3_/hexane 1:1:7) to give the title compound (1.24 g, 93%) as a yellow solid; (m.p. 353.6 K). IR: (KBr) *ν* 3049, 2978, 1718, 1632, 1166, 889, 733, 716 cm^-1^; *δ*_H_ (400 MHz, CDCl_3_) 1.35 (3*H*, t, ^3^*J* = 7.2 Hz), 4.31 (2*H*, q, ^3^*J* = 7.2 Hz, OCH_2_), 6.36 (1*H*, d, ^3^*J* = 16.0 Hz), 7.43 (4*H*, m), 7.95 (2*H*, m), 8.17 (2*H*, m), 8.39 (1*H*, s), 8.57 (1*H*, d, ^3^*J* = 16.0 Hz); *δ*_C_ (100.5 MHz, CDCl_3_) 14.5, 61.0, 125.2, 125.4, 127.2, 128.2, 128.8, 129.3, 129.4, 131.2, 141.9, 166.5; MS: Found: 299.1040 (C_19_H_16_O_2_+Na)^+^; Calcd. for C_19_H_16_O_2_Na: 299.1048. Crystals were grown from cold 2-propanol.

### Refinement

All carbon-bound hydrogen atoms were placed in calculated positions with C—H distances of 0.95 - 1.00 Å and refined as riding with *U*_iso_(H) =*xU*_eq_(C), where *x* = 1.5 for methyl and *x* = 1.2 for all other H-atoms.

### Crystal data

**Table:**

C_19_H_16_O_2_	*F*(000) = 584
*M**_r_* = 276.32	*D*_x_ = 1.306 Mg m^−^^3^
Triclinic, *P*1	Melting point: 353.6 K
*a* = 8.8700 (5) Å	Cu *K*α radiation, λ = 1.5418 Å
*b* = 12.8918 (7) Å	Cell parameters from 4999 reflections
*c* = 13.1062 (7) Å	θ = 3.6–76.1°
α = 84.389 (4)°	µ = 0.66 mm^−^^1^
β = 84.620 (4)°	*T* = 291 K
γ = 70.771 (5)°	Block, translucent intense yellow
*V* = 1405.28 (13) Å^3^	0.22 × 0.11 × 0.09 mm
*Z* = 4	

### Data collection

**Table:**

Agilent SuperNova Dual Atlas diffractometer	4901 independent reflections
Radiation source: SuperNova (Cu) X-ray Source	4250 reflections with *I* > 2σ(*I*)
Mirror monochromator	*R*_int_ = 0.025
Detector resolution: 10.4127 pixels mm^-1^	θ_max_ = 66.0°, θ_min_ = 3.6°
ω scans	*h* = −10→10
Absorption correction: gaussian (*CrysAlis PRO*; Agilent, 2012)	*k* = −10→15
*T*_min_ = 0.889, *T*_max_ = 0.942	*l* = −15→15
11350 measured reflections	

### Refinement

**Table:**

Refinement on *F*^2^	Primary atom site location: structure-invariant direct methods
Least-squares matrix: full	Secondary atom site location: difference Fourier map
*R*[*F*^2^ > 2σ(*F*^2^)] = 0.057	Hydrogen site location: inferred from neighbouring sites
*wR*(*F*^2^) = 0.171	H-atom parameters constrained
*S* = 1.10	*w* = 1/[σ^2^(*F*_o_^2^) + (0.0665*P*)^2^ + 2.0996*P*] where *P* = (*F*_o_^2^ + 2*F*_c_^2^)/3
4901 reflections	(Δ/σ)_max_ = 0.001
381 parameters	Δρ_max_ = 0.29 e Å^−^^3^
0 restraints	Δρ_min_ = −0.26 e Å^−^^3^

### Special details

**Table:**

Geometry. All e.s.d.'s (except the e.s.d. in the dihedral angle between two l.s. planes) are estimated using the full covariance matrix. The cell e.s.d.'s are taken into account individually in the estimation of e.s.d.'s in distances, angles and torsion angles; correlations between e.s.d.'s in cell parameters are only used when they are defined by crystal symmetry. An approximate (isotropic) treatment of cell e.s.d.'s is used for estimating e.s.d.'s involving l.s. planes.
Refinement. Refinement of *F*^2^ against ALL reflections. The weighted *R*-factor *wR* and goodness of fit *S* are based on *F*^2^, conventional *R*-factors *R* are based on *F*, with *F* set to zero for negative *F*^2^. The threshold expression of *F*^2^ > σ(*F*^2^) is used only for calculating *R*-factors(gt) *etc*. and is not relevant to the choice of reflections for refinement. *R*-factors based on *F*^2^ are statistically about twice as large as those based on *F*, and *R*- factors based on ALL data will be even larger.

### Fractional atomic coordinates and isotropic or equivalent isotropic displacement parameters (Å^2^)

**Table:**

	*x*	*y*	*z*	*U*_iso_*/*U*_eq_	
C10A	1.2110 (3)	0.6510 (2)	1.14911 (19)	0.0233 (6)	
C10B	0.5292 (3)	0.5977 (2)	0.65022 (19)	0.0226 (5)	
C11A	1.2162 (3)	0.7552 (2)	1.1299 (2)	0.0248 (6)	
C11B	0.6625 (3)	0.6286 (2)	0.6358 (2)	0.0252 (6)	
C12A	1.0823 (3)	0.8399 (2)	1.0900 (2)	0.0247 (6)	
C12B	0.8112 (3)	0.5498 (2)	0.60494 (19)	0.0232 (5)	
C13A	0.9467 (3)	0.8181 (2)	1.07279 (19)	0.0217 (5)	
C13B	0.8205 (3)	0.4465 (2)	0.58699 (18)	0.0211 (5)	
C14A	0.9348 (3)	0.7097 (2)	1.09399 (18)	0.0185 (5)	
C14B	0.6822 (3)	0.4108 (2)	0.59782 (18)	0.0188 (5)	
C15A	0.6563 (3)	0.7738 (2)	1.03919 (19)	0.0189 (5)	
C15B	0.8340 (3)	0.2220 (2)	0.53924 (19)	0.0192 (5)	
C16A	0.5756 (3)	0.7705 (2)	0.95986 (19)	0.0195 (5)	
C16B	0.9259 (3)	0.2403 (2)	0.45797 (19)	0.0192 (5)	
C17A	0.4367 (3)	0.8650 (2)	0.92781 (19)	0.0187 (5)	
C17B	1.0714 (3)	0.1530 (2)	0.42227 (19)	0.0186 (5)	
C18A	0.2639 (3)	0.9408 (2)	0.7928 (2)	0.0241 (6)	
C18B	1.2700 (3)	0.1079 (2)	0.2833 (2)	0.0253 (6)	
C19A	0.2370 (3)	0.9096 (2)	0.6907 (2)	0.0287 (6)	
C19B	1.2224 (4)	0.0354 (3)	0.2187 (2)	0.0381 (7)	
C1A	0.7963 (3)	0.6843 (2)	1.07686 (18)	0.0179 (5)	
C1B	0.6856 (3)	0.3039 (2)	0.57936 (18)	0.0192 (5)	
C2A	0.7926 (3)	0.5751 (2)	1.09581 (18)	0.0182 (5)	
C2B	0.5461 (3)	0.2731 (2)	0.59795 (18)	0.0187 (5)	
C3A	0.6545 (3)	0.5437 (2)	1.08596 (18)	0.0205 (5)	
C3B	0.5413 (3)	0.1676 (2)	0.57712 (19)	0.0218 (5)	
C4A	0.6582 (3)	0.4370 (2)	1.10317 (19)	0.0238 (5)	
C4B	0.4033 (3)	0.1420 (2)	0.5929 (2)	0.0242 (5)	
C5A	0.7991 (3)	0.3526 (2)	1.1330 (2)	0.0257 (6)	
C5B	0.2589 (3)	0.2189 (2)	0.6321 (2)	0.0234 (5)	
C6A	0.9315 (3)	0.3792 (2)	1.14713 (19)	0.0234 (5)	
C6B	0.2578 (3)	0.3206 (2)	0.65261 (19)	0.0218 (5)	
C7A	0.9337 (3)	0.4898 (2)	1.13063 (18)	0.0208 (5)	
C7B	0.3988 (3)	0.3521 (2)	0.63505 (18)	0.0191 (5)	
C8A	1.0680 (3)	0.5168 (2)	1.14721 (18)	0.0213 (5)	
C8B	0.3970 (3)	0.4574 (2)	0.65117 (19)	0.0213 (5)	
C9A	1.0724 (3)	0.6240 (2)	1.13095 (18)	0.0195 (5)	
C9B	0.5332 (3)	0.4892 (2)	0.63321 (18)	0.0197 (5)	
H10A	1.2994	0.5960	1.1746	0.028*	
H10B	0.4327	0.6487	0.6717	0.027*	
H11A	1.3071	0.7713	1.1428	0.030*	
H11B	0.6569	0.7002	0.6460	0.030*	
H12A	1.0872	0.9109	1.0755	0.030*	
H12B	0.9033	0.5700	0.5970	0.028*	
H13A	0.8604	0.8746	1.0468	0.026*	
H13B	0.9193	0.3970	0.5670	0.025*	
H15A	0.6218	0.8380	1.0739	0.023*	
H15B	0.8655	0.1523	0.5735	0.023*	
H16A	0.6070	0.7074	0.9237	0.023*	
H16B	0.8981	0.3095	0.4229	0.023*	
H18A	1.3367	0.0627	0.3362	0.030*	
H18B	1.3320	0.1471	0.2407	0.030*	
H18C	0.2929	1.0076	0.7845	0.029*	
H18D	0.1673	0.9538	0.8379	0.029*	
H19A	1.1467	0.0803	0.1714	0.057*	
H19B	1.1747	−0.0112	0.2622	0.057*	
H19C	1.3155	−0.0092	0.1811	0.057*	
H19D	0.3357	0.8908	0.6489	0.043*	
H19E	0.1588	0.9705	0.6572	0.043*	
H19F	0.1991	0.8474	0.7005	0.043*	
H3A	0.5599	0.5974	1.0674	0.025*	
H3B	0.6342	0.1153	0.5523	0.026*	
H4A	0.5668	0.4192	1.0952	0.029*	
H4B	0.4034	0.0730	0.5777	0.029*	
H5A	0.8009	0.2798	1.1428	0.031*	
H5B	0.1661	0.1997	0.6436	0.028*	
H6A	1.0229	0.3239	1.1681	0.028*	
H6B	0.1635	0.3706	0.6785	0.026*	
H8A	1.1580	0.4614	1.1700	0.026*	
H8B	0.3018	0.5082	0.6747	0.026*	
O1A	0.3710 (2)	0.94528 (14)	0.97524 (14)	0.0244 (4)	
O1B	1.1304 (2)	0.06439 (14)	0.46678 (14)	0.0241 (4)	
O2A	0.3930 (2)	0.84993 (14)	0.83614 (13)	0.0209 (4)	
O2B	1.1301 (2)	0.18662 (14)	0.33104 (13)	0.0225 (4)	

### Atomic displacement parameters (Å^2^)

**Table:**

	*U*^11^	*U*^22^	*U*^33^	*U*^12^	*U*^13^	*U*^23^
C10A	0.0158 (12)	0.0318 (14)	0.0185 (12)	−0.0022 (10)	−0.0003 (9)	−0.0036 (10)
C10B	0.0230 (13)	0.0200 (12)	0.0203 (12)	−0.0012 (10)	−0.0014 (10)	−0.0012 (10)
C11A	0.0168 (12)	0.0328 (14)	0.0258 (13)	−0.0079 (11)	0.0003 (10)	−0.0077 (11)
C11B	0.0307 (14)	0.0218 (13)	0.0236 (13)	−0.0092 (11)	−0.0030 (11)	−0.0001 (10)
C12A	0.0240 (13)	0.0235 (13)	0.0272 (13)	−0.0084 (11)	0.0023 (11)	−0.0064 (10)
C12B	0.0239 (13)	0.0269 (13)	0.0207 (12)	−0.0110 (11)	−0.0018 (10)	−0.0005 (10)
C13A	0.0178 (12)	0.0215 (12)	0.0227 (12)	−0.0022 (10)	0.0005 (10)	−0.0030 (10)
C13B	0.0191 (12)	0.0263 (13)	0.0164 (12)	−0.0057 (10)	−0.0007 (9)	0.0000 (10)
C14A	0.0174 (12)	0.0194 (12)	0.0155 (11)	−0.0019 (9)	0.0009 (9)	−0.0027 (9)
C14B	0.0185 (12)	0.0202 (12)	0.0149 (11)	−0.0022 (10)	−0.0020 (9)	−0.0010 (9)
C15A	0.0156 (11)	0.0167 (11)	0.0228 (12)	−0.0040 (9)	0.0015 (9)	−0.0005 (9)
C15B	0.0170 (12)	0.0186 (12)	0.0214 (12)	−0.0048 (9)	−0.0025 (9)	−0.0007 (9)
C16A	0.0166 (11)	0.0178 (12)	0.0231 (12)	−0.0049 (9)	0.0019 (9)	−0.0025 (9)
C16B	0.0170 (12)	0.0176 (12)	0.0214 (12)	−0.0035 (9)	−0.0024 (9)	−0.0005 (9)
C17A	0.0167 (11)	0.0187 (12)	0.0214 (12)	−0.0079 (10)	0.0006 (9)	0.0003 (10)
C17B	0.0162 (11)	0.0204 (12)	0.0202 (12)	−0.0068 (10)	−0.0007 (9)	−0.0031 (10)
C18A	0.0193 (12)	0.0204 (13)	0.0289 (14)	−0.0016 (10)	−0.0061 (10)	0.0026 (10)
C18B	0.0190 (12)	0.0261 (13)	0.0265 (13)	−0.0037 (10)	0.0081 (10)	−0.0042 (11)
C19A	0.0302 (14)	0.0285 (14)	0.0266 (14)	−0.0084 (12)	−0.0083 (11)	0.0044 (11)
C19B	0.0404 (17)	0.0382 (17)	0.0340 (16)	−0.0102 (14)	0.0088 (13)	−0.0137 (13)
C1A	0.0163 (11)	0.0180 (12)	0.0161 (11)	−0.0013 (9)	0.0014 (9)	−0.0028 (9)
C1B	0.0186 (12)	0.0218 (12)	0.0144 (11)	−0.0033 (10)	−0.0017 (9)	0.0010 (9)
C2A	0.0181 (12)	0.0191 (12)	0.0137 (11)	−0.0016 (10)	0.0008 (9)	−0.0006 (9)
C2B	0.0176 (12)	0.0210 (12)	0.0149 (11)	−0.0027 (10)	−0.0016 (9)	0.0001 (9)
C3A	0.0194 (12)	0.0221 (13)	0.0176 (12)	−0.0037 (10)	0.0002 (9)	−0.0011 (9)
C3B	0.0189 (12)	0.0218 (13)	0.0219 (12)	−0.0035 (10)	0.0012 (10)	−0.0017 (10)
C4A	0.0279 (13)	0.0266 (13)	0.0187 (12)	−0.0119 (11)	0.0018 (10)	−0.0026 (10)
C4B	0.0252 (13)	0.0214 (13)	0.0257 (13)	−0.0080 (11)	−0.0005 (10)	−0.0005 (10)
C5A	0.0322 (14)	0.0194 (12)	0.0239 (13)	−0.0075 (11)	0.0039 (11)	−0.0021 (10)
C5B	0.0178 (12)	0.0277 (13)	0.0246 (13)	−0.0081 (10)	−0.0006 (10)	0.0005 (10)
C6A	0.0253 (13)	0.0190 (12)	0.0198 (12)	−0.0002 (10)	0.0013 (10)	0.0009 (10)
C6B	0.0168 (12)	0.0252 (13)	0.0198 (12)	−0.0027 (10)	−0.0003 (9)	−0.0004 (10)
C7A	0.0213 (12)	0.0213 (12)	0.0150 (11)	−0.0017 (10)	0.0029 (9)	−0.0008 (9)
C7B	0.0170 (12)	0.0226 (12)	0.0145 (11)	−0.0025 (10)	−0.0005 (9)	−0.0003 (9)
C8A	0.0175 (12)	0.0223 (12)	0.0174 (12)	0.0020 (10)	0.0005 (9)	−0.0004 (9)
C8B	0.0182 (12)	0.0215 (12)	0.0179 (12)	0.0023 (10)	−0.0003 (9)	−0.0024 (9)
C9A	0.0169 (12)	0.0228 (12)	0.0158 (11)	−0.0026 (10)	0.0012 (9)	−0.0022 (9)
C9B	0.0200 (12)	0.0220 (12)	0.0143 (11)	−0.0028 (10)	−0.0034 (9)	0.0001 (9)
O1A	0.0202 (9)	0.0223 (9)	0.0282 (10)	−0.0021 (7)	−0.0034 (7)	−0.0047 (7)
O1B	0.0199 (9)	0.0215 (9)	0.0264 (9)	−0.0022 (7)	0.0016 (7)	0.0017 (7)
O2A	0.0182 (8)	0.0202 (9)	0.0213 (9)	−0.0021 (7)	−0.0036 (7)	0.0004 (7)
O2B	0.0188 (9)	0.0225 (9)	0.0222 (9)	−0.0033 (7)	0.0046 (7)	−0.0006 (7)

### Geometric parameters (Å, º)

**Table:**

C10A—C11A	1.358 (4)	C1A—C15A	1.476 (3)
C10A—H10A	0.9300	C1A—C14A	1.414 (4)
C10B—H10B	0.9300	C1A—C2A	1.415 (4)
C11A—H11A	0.9300	C1B—C15B	1.479 (3)
C11B—C10B	1.359 (4)	C2A—C3A	1.432 (4)
C11B—H11B	0.9300	C2A—C7A	1.445 (3)
C12A—C11A	1.424 (4)	C2B—C3B	1.429 (4)
C12A—H12A	0.9300	C2B—C7B	1.443 (3)
C12B—C11B	1.426 (4)	C2B—C1B	1.413 (4)
C12B—C13B	1.350 (4)	C3A—H3A	0.9300
C12B—H12B	0.9300	C3B—H3B	0.9300
C13A—C12A	1.364 (4)	C4A—C5A	1.418 (4)
C13A—H13A	0.9300	C4A—C3A	1.362 (4)
C13B—H13B	0.9300	C4A—H4A	0.9300
C14A—C13A	1.434 (4)	C4B—C3B	1.362 (4)
C14A—C9A	1.436 (3)	C4B—C5B	1.424 (4)
C14B—C13B	1.436 (4)	C4B—H4B	0.9300
C14B—C9B	1.442 (3)	C5A—H5A	0.9300
C14B—C1B	1.412 (4)	C5B—H5B	0.9300
C15A—C16A	1.328 (4)	C6A—C5A	1.360 (4)
C15A—H15A	0.9300	C6A—H6A	0.9300
C15B—H15B	0.9300	C6B—C5B	1.361 (4)
C16A—C17A	1.478 (3)	C6B—H6B	0.9300
C16A—H16A	0.9300	C7A—C6A	1.427 (4)
C16B—C17B	1.478 (3)	C7B—C8B	1.389 (4)
C16B—C15B	1.330 (4)	C7B—C6B	1.429 (4)
C16B—H16B	0.9300	C8A—C7A	1.387 (4)
C18A—C19A	1.497 (4)	C8A—H8A	0.9300
C18A—H18D	0.9700	C8B—H8B	0.9300
C18A—H18C	0.9700	C9A—C10A	1.428 (4)
C18B—C19B	1.500 (4)	C9A—C8A	1.391 (4)
C18B—H18B	0.9700	C9B—C10B	1.427 (4)
C18B—H18A	0.9700	C9B—C8B	1.392 (4)
C19A—H19F	0.9600	O1A—C17A	1.207 (3)
C19A—H19E	0.9600	O1B—C17B	1.205 (3)
C19A—H19D	0.9600	O2A—C18A	1.454 (3)
C19B—H19C	0.9600	O2A—C17A	1.347 (3)
C19B—H19B	0.9600	O2B—C18B	1.453 (3)
C19B—H19A	0.9600	O2B—C17B	1.349 (3)
			
C10A—C11A—H11A	120.0	C2B—C3B—H3B	119.4
C10A—C11A—C12A	120.0 (2)	C2B—C1B—C15B	118.4 (2)
C10A—C9A—C14A	119.1 (2)	C3A—C4A—C5A	121.2 (3)
C10B—C11B—H11B	120.4	C3A—C4A—H4A	119.4
C10B—C11B—C12B	119.1 (2)	C3A—C2A—C7A	117.1 (2)
C10B—C9B—C14B	119.0 (2)	C3B—C4B—C5B	121.3 (2)
C11A—C12A—H12A	119.7	C3B—C4B—H4B	119.4
C11A—C10A—H10A	119.4	C3B—C2B—C7B	117.5 (2)
C11A—C10A—C9A	121.3 (2)	C4A—C5A—H5A	120.3
C11B—C10B—H10B	119.1	C4A—C3A—H3A	119.2
C11B—C10B—C9B	121.8 (2)	C4A—C3A—C2A	121.5 (2)
C11B—C12B—H12B	119.4	C4B—C3B—H3B	119.4
C12A—C11A—H11A	120.0	C4B—C3B—C2B	121.2 (2)
C12A—C13A—H13A	119.4	C4B—C5B—H5B	120.3
C12A—C13A—C14A	121.2 (2)	C5A—C6A—H6A	119.1
C12B—C11B—H11B	120.4	C5A—C6A—C7A	121.7 (2)
C12B—C13B—H13B	119.1	C5A—C4A—H4A	119.4
C12B—C13B—C14B	121.9 (2)	C5B—C4B—H4B	119.4
C13A—C12A—C11A	120.7 (2)	C5B—C6B—H6B	119.4
C13A—C12A—H12A	119.7	C5B—C6B—C7B	121.2 (2)
C13A—C14A—C9A	117.7 (2)	C6A—C5A—H5A	120.3
C13B—C12B—C11B	121.1 (2)	C6A—C5A—C4A	119.4 (2)
C13B—C12B—H12B	119.4	C6A—C7A—C2A	119.0 (2)
C13B—C14B—C9B	116.9 (2)	C6B—C5B—H5B	120.3
C14A—C13A—H13A	119.4	C6B—C5B—C4B	119.5 (2)
C14A—C1A—C15A	118.6 (2)	C6B—C7B—C2B	119.3 (2)
C14A—C1A—C2A	120.5 (2)	C7A—C6A—H6A	119.1
C14B—C13B—H13B	119.1	C7A—C8A—H8A	118.9
C14B—C1B—C15B	121.1 (2)	C7A—C8A—C9A	122.2 (2)
C14B—C1B—C2B	120.5 (2)	C7B—C8B—H8B	118.9
C15A—C16A—C17A	121.4 (2)	C7B—C8B—C9B	122.1 (2)
C15A—C16A—H16A	119.3	C7B—C6B—H6B	119.4
C15B—C16B—C17B	121.4 (2)	C8A—C7A—C6A	121.5 (2)
C15B—C16B—H16B	119.3	C8A—C7A—C2A	119.5 (2)
C16A—C15A—H15A	117.3	C8A—C9A—C10A	121.7 (2)
C16A—C15A—C1A	125.5 (2)	C8A—C9A—C14A	119.2 (2)
C16B—C15B—H15B	117.5	C8B—C9B—C10B	121.4 (2)
C16B—C15B—C1B	125.0 (2)	C8B—C9B—C14B	119.6 (2)
C17A—C16A—H16A	119.3	C8B—C7B—C6B	121.7 (2)
C17A—O2A—C18A	115.54 (19)	C8B—C7B—C2B	119.1 (2)
C17B—C16B—H16B	119.3	C9A—C10A—H10A	119.4
C17B—O2B—C18B	116.66 (19)	C9A—C8A—H8A	118.9
C18A—C19A—H19F	109.5	C9B—C10B—H10B	119.1
C18A—C19A—H19E	109.5	C9B—C8B—H8B	118.9
C18A—C19A—H19D	109.5	H18A—C18B—H18B	108.0
C18B—C19B—H19C	109.5	H18C—C18A—H18D	108.5
C18B—C19B—H19B	109.5	H19A—C19B—H19C	109.5
C18B—C19B—H19A	109.5	H19A—C19B—H19B	109.5
C19A—C18A—H18D	110.3	H19B—C19B—H19C	109.5
C19A—C18A—H18C	110.3	H19D—C19A—H19F	109.5
C19B—C18B—H18B	109.4	H19D—C19A—H19E	109.5
C19B—C18B—H18A	109.4	H19E—C19A—H19F	109.5
C1A—C15A—H15A	117.3	O1A—C17A—C16A	125.9 (2)
C1A—C14A—C13A	122.7 (2)	O1A—C17A—O2A	123.9 (2)
C1A—C14A—C9A	119.6 (2)	O1B—C17B—C16B	125.9 (2)
C1A—C2A—C3A	123.9 (2)	O1B—C17B—O2B	124.2 (2)
C1A—C2A—C7A	119.0 (2)	O2A—C18A—C19A	107.3 (2)
C1B—C15B—H15B	117.5	O2A—C18A—H18D	110.3
C1B—C2B—C3B	122.9 (2)	O2A—C18A—H18C	110.3
C1B—C2B—C7B	119.6 (2)	O2A—C17A—C16A	110.2 (2)
C1B—C14B—C13B	124.0 (2)	O2B—C18B—C19B	111.0 (2)
C1B—C14B—C9B	119.1 (2)	O2B—C18B—H18B	109.4
C2A—C3A—H3A	119.2	O2B—C18B—H18A	109.4
C2A—C1A—C15A	121.0 (2)	O2B—C17B—C16B	109.9 (2)
			
C10A—C9A—C8A—C7A	179.5 (2)	C1A—C2A—C7A—C8A	1.6 (3)
C10B—C9B—C8B—C7B	−180.0 (2)	C1B—C2B—C3B—C4B	−177.8 (2)
C11B—C12B—C13B—C14B	0.1 (4)	C1B—C2B—C7B—C8B	0.4 (3)
C12B—C11B—C10B—C9B	−1.3 (4)	C1B—C2B—C7B—C6B	179.3 (2)
C13A—C12A—C11A—C10A	1.4 (4)	C1B—C14B—C13B—C12B	179.6 (2)
C13A—C14A—C9A—C10A	2.7 (3)	C1B—C14B—C9B—C10B	−179.0 (2)
C13A—C14A—C9A—C8A	−176.7 (2)	C1B—C14B—C9B—C8B	1.8 (3)
C13B—C12B—C11B—C10B	1.9 (4)	C2A—C7A—C6A—C5A	−1.4 (4)
C13B—C14B—C9B—C10B	3.1 (3)	C2A—C1A—C15A—C16A	−49.8 (4)
C13B—C14B—C9B—C8B	−176.1 (2)	C2A—C1A—C14A—C13A	177.9 (2)
C13B—C14B—C1B—C15B	−4.6 (4)	C2A—C1A—C14A—C9A	0.0 (3)
C13B—C14B—C1B—C2B	176.0 (2)	C2B—C7B—C8B—C9B	−0.3 (4)
C14A—C13A—C12A—C11A	−0.1 (4)	C2B—C7B—C6B—C5B	−2.0 (4)
C14A—C9A—C10A—C11A	−1.5 (4)	C2B—C1B—C15B—C16B	129.6 (3)
C14A—C9A—C8A—C7A	−1.1 (4)	C3A—C4A—C5A—C6A	1.6 (4)
C14A—C1A—C15A—C16A	130.4 (3)	C3A—C2A—C7A—C6A	3.7 (3)
C14A—C1A—C2A—C3A	176.4 (2)	C3A—C2A—C7A—C8A	−176.3 (2)
C14A—C1A—C2A—C7A	−1.4 (3)	C3B—C4B—C5B—C6B	1.1 (4)
C14B—C9B—C10B—C11B	−1.2 (4)	C3B—C2B—C7B—C8B	−176.8 (2)
C14B—C9B—C8B—C7B	−0.7 (4)	C3B—C2B—C7B—C6B	2.2 (3)
C14B—C1B—C15B—C16B	−49.8 (4)	C3B—C2B—C1B—C15B	−1.7 (4)
C15A—C16A—C17A—O2A	169.2 (2)	C3B—C2B—C1B—C14B	177.7 (2)
C15A—C16A—C17A—O1A	−10.2 (4)	C5A—C4A—C3A—C2A	0.9 (4)
C15A—C1A—C14A—C13A	−2.3 (4)	C5B—C4B—C3B—C2B	−0.9 (4)
C15A—C1A—C14A—C9A	179.8 (2)	C6B—C7B—C8B—C9B	−179.3 (2)
C15A—C1A—C2A—C3A	−3.5 (4)	C7A—C6A—C5A—C4A	−1.3 (4)
C15A—C1A—C2A—C7A	178.7 (2)	C7A—C2A—C3A—C4A	−3.5 (3)
C15B—C16B—C17B—O2B	171.2 (2)	C7B—C6B—C5B—C4B	0.3 (4)
C15B—C16B—C17B—O1B	−8.7 (4)	C7B—C2B—C3B—C4B	−0.8 (4)
C17A—O2A—C18A—C19A	178.4 (2)	C7B—C2B—C1B—C15B	−178.7 (2)
C17B—C16B—C15B—C1B	−179.1 (2)	C7B—C2B—C1B—C14B	0.7 (4)
C17B—O2B—C18B—C19B	87.3 (3)	C8A—C7A—C6A—C5A	178.6 (2)
C18A—O2A—C17A—C16A	−176.1 (2)	C8A—C9A—C10A—C11A	177.9 (2)
C18A—O2A—C17A—O1A	3.3 (3)	C8B—C9B—C10B—C11B	178.0 (2)
C18B—O2B—C17B—C16B	−177.5 (2)	C8B—C7B—C6B—C5B	176.9 (2)
C18B—O2B—C17B—O1B	2.4 (4)	C9A—C10A—C11A—C12A	−0.6 (4)
C1A—C15A—C16A—C17A	−179.8 (2)	C9A—C8A—C7A—C6A	179.6 (2)
C1A—C14A—C13A—C12A	−179.9 (2)	C9A—C8A—C7A—C2A	−0.3 (4)
C1A—C14A—C9A—C10A	−179.3 (2)	C9A—C14A—C13A—C12A	−1.9 (4)
C1A—C14A—C9A—C8A	1.3 (3)	C9B—C14B—C13B—C12B	−2.6 (4)
C1A—C2A—C3A—C4A	178.7 (2)	C9B—C14B—C1B—C15B	177.6 (2)
C1A—C2A—C7A—C6A	−178.4 (2)	C9B—C14B—C1B—C2B	−1.7 (3)

### Hydrogen-bond geometry (Å, º)

Cg1 and Cg2 are the centroids of the C1*A*/C2*A*/C7*A*–C9*A*/C14*A* and C2*A*—C7*A* rings, respectively.

**Table:**

*D*—H···*A*	*D*—H	H···*A*	*D*···*A*	*D*—H···*A*
C13*A*—H13*A*···O1*A*^i^	0.93	2.56	3.455 (3)	163
C18*B*—H18*B*···O2*A*^ii^	0.97	2.56	3.422 (3)	148
C3*B*—H3*B*···O1*B*^iii^	0.93	2.57	3.470 (3)	162
C6*A*—H6*A*···O2*B*^iv^	0.93	2.67	3.438 (3)	140
C19*A*—H19*E*···O1*B*^v^	0.96	2.66	3.409 (3)	135
C6*B*—H6*B*···*Cg*1^vi^	0.93	2.81	3.447 (3)	126
C8*B*—H8*B*···*Cg*2^vi^	0.93	2.82	3.439 (3)	124

Symmetry codes: (i) −*x*+1, −*y*+2, −*z*+2; (ii) −*x*+2, −*y*+1, −*z*+1; (iii) −*x*+2, −*y*, −*z*+1; (iv) *x*, *y*, *z*+1; (v) *x*−1, *y*+1, *z*; (vi) −*x*+1, −*y*+1, −*z*+2.
